# Can hydration protect against intravenous contrast-induced acute kidney injury?

**DOI:** 10.1590/0100-3984.2025.0017-en

**Published:** 2025-11-28

**Authors:** Cassiane Dezoti da Fonseca, Dayse Santana Santos, Eduesley Santana Santos, Clara Versolato Razvickas, Bianca Castino, Fernanda Teixeira Borges, Maria de Fatima Fernandes Vattimo

**Affiliations:** 1 Universidade Federal de São Paulo (Unifesp), São Paulo, SP, Brazil.; 2 Universidade Federal de Sergipe (UFS), São Cristóvão, SE, Brazil.; 3 Universidade Cruzeiro do Sul, São Paulo, SP, Brazil.; 4 Universidade de São Paulo (USP), São Paulo, SP, Brazil.

**Keywords:** Diabetes mellitus, Kidney/drug effects, Kidney diseases/chemically induced, Contrast media, Risk factors, Primary prevention, Diabetes mellitus, Rim/efeitos dos fármacos, Meios de contraste, Fatores de risco, Prevenção primária

## Abstract

**Objective:**

To evaluate the effect of saline hydration on contrast-induced acute kidney injury in a rat model of diabetes mellitus.

**Materials and Methods:**

This was a quantitative, preclinical experimental study. A total of 28 male Wistar rats were randomized into four groups: citrate (control); diabetes mellitus-only; diabetes mellitus + iodinated contrast (6 mL/kg iothalamate meglumine); and diabetes mellitus + iodinated contrast + saline (NaCl 0.9%, 12 mL/kg). Physiological parameters, renal hemodynamics, inulin clearance (as a proxy for renal function), urinary albumin, and oxidative injury were assessed. Statistical significance was set at *p* < 0.05. Results: In the diabetes mellitus-only group, there was sustained hyperglycemia, weight loss, polyphagia, polyuria, polydipsia, and renal hypertrophy, with significant differences in comparison with the control group. In the diabetes mellitus + iodinated contrast group (in comparison with the diabetes mellitus-only group), there was an additional reduction in the mean renal blood flow (2.1 ± 0.7 mL/min vs. 6.9 ± 0.8 mL/min), greater mean renal vascular resistance, lower mean inulin clearance (0.17 ± 0.02 mL/min vs. 0.85 ± 0.13 mL/min), and a higher mean level of urinary neutrophil gelatinase-associated lipocalin (318.1 ± 52.6 pg/mL vs. 42.2 ± 42.6 pg/mL), together with higher hydrogen peroxide concentrations, as well as elevated lipid peroxidation and thiol consumption in renal tissue. Pretreatment with saline hydration averted those changes (*p* < 0.05 for all).

**Conclusion:**

Saline hydration attenuated the impairment of renal function and hemodynamics by reducing redox imbalance in contrast-induced acute kidney injury.

## Abstract (pt)

**Objetivo:**

Avaliar o efeito da hidratação salina na injúria renal aguda induzida por contraste no fator de risco diabetes mellitus em ratos Wistar machos.

**Materiais e Métodos:**

Estudo experimental quantitativo e pré-clínico. Um total de 28 animais foi randomizado em quatro grupos: citrato-controle; diabetes mellitus; diabetes mellitus + contraste iodado (6 mL/kg de ioxatalamato de meglumina); diabetes mellitus + contraste iodado + soro fisiológico (NaCl 0,9%, 12 mL/kg). Foram avaliados parâmetros fisiológicos, hemodinâmica renal, função renal, albumina urinária e lesão oxidativa. A significância estatística foi definida em *p* < 0,05.

**Resultados:**

Foram observadas hiperglicemia sustentada, perda de peso, polifagia, poliúria, polidipsia e hipertrofia renal no grupo diabetes mellitus. O contraste iodado contribuiu para a redução do fluxo sanguíneo renal adicional (2,1 ± 0,7 mL/min vs. 6,9 ± 0,8 mL/min), aumento da resistência vascular renal, diminuição do *clearance* de inulina (0,17 ± 0,02 mL/min vs. 0,85 ± 0,13 mL/ min) e aumento na lipocalina associada a gelatinase neutrofílica urinária (318,1 ± 52,6 pg/mL vs. 42,2 ± 42,6 pg/mL), além da elevação da concentração de peróxido de hidrogênio, aumento da peroxidação lipídica e do consumo de tiol no tecido renal. O pré- tratamento com a hidratação salina reverteu esses parâmetros (*p* < 0,05).

**Conclusão:**

A hidratação salina atenuou o comprometimento da função e da hemodinâmica renais, ao reduzir o desequilíbrio *redox* na injúria renal aguda induzida por contraste.

## INTRODUCTION

In recent years, the use of iodine-based contrast agents as contrast-induced acute kidney injury (CI-AKI) have in computed tomography and angiographic examinations emerged as significant complications in radiology clinical has become an indispensable tool in the clinical diagnosis practice^**(1)**^. In various settings, particularly during coronary of various diseases. In this context, adverse events such interventions, CI-AKI is considered an iatrogenic event.

According to Mehran et al.^**(1)**^ and the Kidney Disease: Improving Global Outcomes acute kidney injury work group^**(2)**^, CI-AKI is defined as an increase in serum creatinine of ≥ 25% or ≥ 0.5 mg/dL within the first 72 h after exposure to iodinated contrast media^**(1,2)**^.

The incidence of CI-AKI is 2–12% among presumably euvolemic patients and can be up to 50% among patients with risk factors^**(3)**^. Diabetes mellitus (DM) is considered an important risk factor because sustained hyperglycemia can be mediated by activation of endothelin and the hypersensitivity of renal vessels to adenosine, which together result in vasoconstriction^**(4,5)**^. In addition, the use of iodinated contrast induces direct cellular toxicity that favors the formation of reactive oxygen species through enzymatic and non-enzymatic mechanisms that include the Fenton reaction catalyzed by unbound iron and the endogenous consumption of antioxidants^**(1)**^.

Intravenous or oral hydration is an appropriate, safe measure that is universally accepted^**(3,6)**^. Chien et al.^**(7)**^ demonstrated that pretreatment with 4 mL/kg of 0.9% NaCl was renoprotective in CI-AKI due to gadolinium exposure. In addition, a preclinical study of CI-AKI envisioned pretreatment with intravenous saline solution and oral hydration, the combination of which was found to improve renal function and tissue recovery^**(8)**^.

Given that CI-AKI is a potentially preventable adverse event and needs to be considered in patient safety protocols, the aim of the present study was to elucidate the hemodynamics and oxidative mechanisms involved in CIAKI in the context of DM as a risk factor, as well as to assess saline hydration as a low-cost alternative for treatment of the condition.

## MATERIALS AND METHODS

### Study design

Adult male Wistar rats were obtained from the Center for the Development of Experimental Models for Medicine and Biology, in the city of São Paulo, Brazil, and housed in transparent polycarbonate cages. The animals were maintained in a temperature- and humidity-controlled environment (24°C and 60% relative humidity), on a 12/12-h light/dark cycle, with free access to water and rat chow (Nuvilab CR-1; Nuvital Nutrientes Ltda., Colombo, Brazil). They were randomized into four groups (n = 7 per group): the citrate (control) group, in which citrate buffer was administered intravenously (0.01 M, pH 4.2, into the caudal vein) on day 1, after which the animals were maintained in controlled conditions for four weeks; the DM-only group, in which DM was induced by intravenous administration of streptozotocin (65 mg/kg, diluted in 0.01 M citrate buffer, pH 4.2) on day 1^**(4)**^, after which the animals were maintained in controlled conditions for four weeks; the DM + iodinated contrast (DMIC) group, in which DM was induced as in the DM group and the animals received an intraperitoneal injection of iothalamate meglumine (6 mL/kg) on day 26^**(9)**^; and the DM + iodinated contrast + saline hydration (DMICSH) group, in which DM was induced as in the DM group and the animals were treated with intraperitoneal saline hydration (NaCl 0.9%, 12 mL/kg) from day 23 to day 28^**(7)**^; that is, before and after receiving iodinated contrast on day 26. Because this was an experimental study, the comparison between groups allowed us to isolate the effects that DM, contrast administration, and hydration each have on renal function. Given that the animals were randomly allocated and shared the same genetic background and age range, initial homogeneity among the groups was assumed. The procedures were conducted in accordance with the Ethical Principles of Animal Experimentation adopted by the Brazilian College of Animal Experimentation. The study was approved by the Ethics Committees on the Use of Animals of the Federal University of São Paulo (Reference no. 4438210119) and of the Nursing School of the University of São Paulo (Reference no. 1269/2019).

### Blood sample collection and euthanasia

On day 28 of the protocol, at 48 h after contrast administration, the animals were placed in metabolic cages for 24-h urine collection. On day 29, at 72 h after contrast administration, the rats were anesthetized with intraperitoneal injections of xylazine (10 mg/kg) and ketamine (90 mg/kg), thereafter undergoing a surgical procedure to measure renal function and hemodynamics. A blood sample was then collected through abdominal aorta puncture, and the kidneys were prepared for quantification of antioxidant enzymes. The rats were euthanized by physical exsanguination, in accordance with the ethical standards for animal experimentation^**(10)**^.

### Determination of renal hemodynamics

Renal blood flow (RBF) was measured with an ultrasonic flow meter (T402; Transonic Systems Inc., Ithaca, NY, USA) placed around the isolated renal artery. To determine renal vascular resistance (RVR), mean arterial pressure (MAP) and RBF were measured through a PE-60 catheter inserted into the left carotid artery. The RVR was calculated with the following formula^**(11)**^:

RVR=MAP/RBF

### Quantification of renal function

Renal function was assessed by measuring inulin clearance (mL/min). Rats received 100 mg/kg of body weight of inulin solution (20 mg/mL), followed by continuous infusion of 0.04 mL/min of inulin solution (6 mg/mL) into the right jugular vein through a PE-60 catheter. After a 30-min equilibration period, three urine samples were collected through the bladder catheter, and two blood samples were obtained from the carotid artery catheter. Serum and urinary inulin were measured with the anthrone method^**(12)**^. Serum creatinine concentrations were measured with the Jaffe method^**(13)**^. Elevated urinary neutrophil gelatinaseassociated lipocalin (NGAL), which is considered an early biomarker of CI-AKI, has high specificity for tubular injury and occurs 2–4 h after contrast infusion, unlike the rise in creatinine, which occurs 24–72 h after contrast infusion. We analyzed NGAL with a commercially available kit (Rat-NGAL ELISA kit; BioVendor R&D, Brno, Czech Republic). Albumin concentrations in 24-h urine samples were also assessed with a commercially available kit (Rat Albumin ELISA kit; Bethyl Laboratories, Montgomery, TX, USA). Both kits were employed as previously described^**(9)**^.

### Determination of the oxidative profile

Urinary peroxides are biomarkers of oxidative stress, and their elevated concentrations in urine may indicate renal tubular injury. In this study, urinary peroxides were determined with the ferrous oxidation-xylenol orange method, version 2, and the values were corrected for urinary creatinine^**(14)**^. Glutathione is present in all cells and constitutes the main redox buffer, its biological functions being centered on the thiol group. To complement the assessment of oxidative stress, thiols were analyzed according to the following principle: the greater the degree of oxidative stress is, the higher will be the levels of oxidized thiols and the lower will be the concentration of thiols in renal tissue. Non-protein soluble thiols in the kidney were assessed by tissue homogenization in 1 mL of a solution containing 10 mM sodium acetate, 0.5% Tween 20, and 100 µM DTPA (pH 6.5). The thiols were quantified on the basis of their mean absorbance at 412 nm^**(15)**^. Malondialdehyde is a byproduct of the oxidative chain after lipid peroxidation of the cell membrane and is considered a biomarker of oxidative stress. Lipid peroxidation levels of malondialdehyde were determined by measuring thiobarbituric acid reactive substances (TBARS). To quantify peroxidation, 0.4 mL of a urine sample mixed with 0.6 mL water were added to a reaction mixture consisting of 1.0 mL of 17.5% trichloroacetic acid and 1.0 mL of 0.6% thiobarbituric acid. The solution was read in a spectrophotometer at 535 nm^**(16)**^.

### Statistical analysis

Quantitative data are expressed as mean ± standard error of the mean. One-way analysis of variance of the means was carried out, and confidence intervals were calculated. Pairwise comparisons were made with Tukey’s post hoc test. All statistical analyses were performed with GraphPad Prism, version 8 (GraphPad Software; San Diego, CA, USA). Statistical significance was set at *p* < 0.05.

## RESULTS

### Effects of saline hydration on physiological parameters

**Figure 1:**
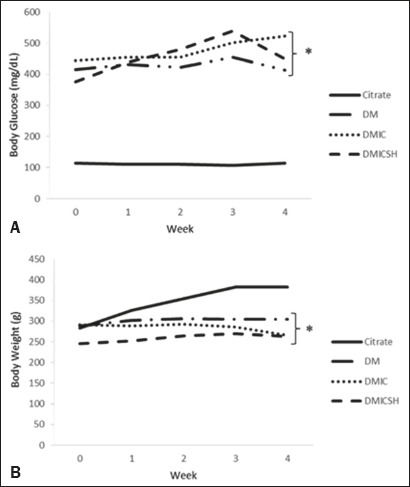
Body glucose and body weight over a four-week period. Data are mean ± standard error of the mean for each group.

Figure 1 shows the mean weekly blood glucose and body weight. In the DM-only, DMIC, and DMICSH groups (i.e., the experimental groups), both of those parameters differed significantly from what was observed for the control group (*p* < 0.05 for all).

Table 1 shows the indicators of physiological parameters such as polydipsia, polyphagia, and polyuria, with greater water intake, food intake, and urine output being observed in the experimental group rats, which also developed kidney hypertrophy (*p* < 0.001). Kidney weight and the kidney weight/body weight ratio were both lower in the DMICSH group than in the DMIC group (*p* < 0.05).

**Table 1:** Physiological parameters before and after CI-AKI, showing the effect of pretreatment with saline hydration.

Group	n	Intake				
		Kidney weight (g)	Kidney weight/Body weight ratio (x 10^–^3)	Food (g/24 h)	Water (mL/24 h)	Urine output (mL/min)
		Mean ± SEM	Mean ± SEM	Mean ± SEM	Mean ± SEM	Mean ± SEM
Control	7	1.39 ± 0.18	0.37 ± 0.04	22 ± 2	28 ± 4	0.012 ± 0.005
DM^–^only	7	1.57 ± 0.15*	0.60 ± 0.05*	33 ± 4*	114 ± 27*	0.046 ± 0.016*
DMIC	7	1.72 ± 0.21*,†	0.69 ± 0.10*	36 ± 5*,†	132 ± 27*	0.053 ± 0.015*
DMICSH	7	1.46 ± 0.10*,‡	0.56 ± 0.07*,‡	57 ± 16*,‡	151 ± 69*	0.070 ± 0.026*

SEM, standard error of the mean.

* *p* < 0.001 vs. control.

† *p* < 0.001 vs. DM.

‡ *p* < 0.001 vs. DMIC.

### Pretreatment with saline hydration attenuated the decrease in RBF after CI-AKI in rats with DM

As can be seen in Table 2, the mean heart rates were significantly higher in the experimental groups than in the control group (*p* < 0.05 for all). In addition, the mean MAP was significantly lower in the DMIC group than in the DM-only group (*p* < 0.05). Furthermore, the RBF was significantly lower in the experimental groups than in the control group (*p* < 0.05 for all). Moreover, the mean RBF was significantly lower in the DMIC group than in the DM-only group (*p* < 0.001), whereas it was significantly higher in the DMICSH group than in the DMIC group (*p* < 0.05). However, the mean RVR was significantly higher in the experimental groups than in the control group (*p* < 0.001 for all), as well as being significantly higher in the DMIC group than in the DM-only group (*p* < 0.001), whereas it was significantly lower in the DMICSH group than in the DMIC group (*p* < 0.05). higher in all three experimental groups than in the control group (*p* < 0.05), an alteration that was mitigated by pretreatment with saline hydration.

**Table 2:** Hemodynamic parameters before and after CI-AKI, showing the effect of pretreatment with saline hydration.

Group	n	Heart rate(bpm) Mean ± SEM	MAP(mmHg) Mean ± SEM	RBF(mL/min) Mean ± SEM	RVR(mmHg/mL/min) Mean ± SEM
Control	7	331 ± 54	83 ± 11	6.9 ± 0.8	10.1 ± 2.3
DM-only	7	453 ± 53*	101 ± 10	4.3 ± 2.0*	26.3 ± 2.3*
DMIC	7	318 ± 69*	77 ± 10t	2.1 ± 0.7*,†	32.0 ± 9.2*,†
DMICSH	7	574 ± 21*,‡,‡	87 ± 14	4.7 ± 1.3*	19.8 ± 5.0*,†,‡

SEM, standard error of the mean.

* *p* < 0.001 vs. control.

† *p* < 0.001 vs. DM.

‡ *p* < 0.001 vs. DMIC.

### Pretreatment with saline hydration attenuated the increase in oxidative stress after CI-AKI in rats with DM

**Figure 2:**
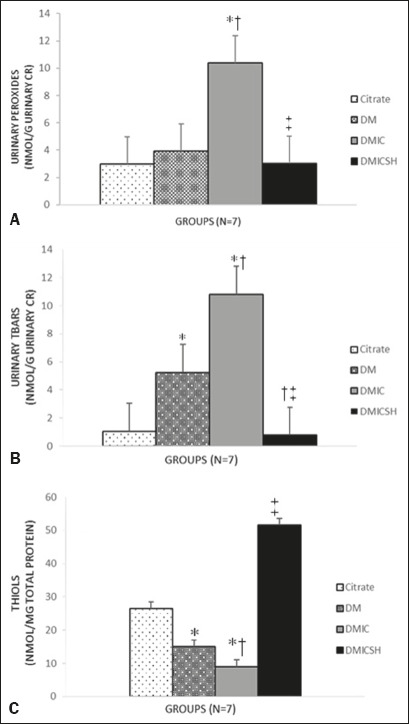
Effect that pretreatment with saline hydration has on the oxidative profile. a: Urinary peroxides. b: Urinary TBARS; c: Thiol levels. CR, creatinine. Data are mean ± standard error of the mean for each group. * *p* < 0.001 vs. control. ^† ^*p* < 0.001 vs. DM-only. ^‡ ^*p* < 0.001 vs. DMIC.

The mean concentration of hydrogen peroxide (in nmol/g creatinine) was higher in the DMIC group than in the control group (11.38 ± 3.77 vs. 2.77 ± 1.20, *p* < 0.05). In the group that received pretreatment with hydration (the DMICSH group), the mean concentration of hydrogen peroxide was lower than it was in the DMIC group (3.02 ± 1.31 vs. 11.38 ± 3.77, *p* < 0.05), as illustrated in Figure 2A. As shown in Figure 2B, the mean TBARS level (in nmol/g creatinine) was higher in the DM-only group

### Pretreatment with saline hydration attenuated the impairment of renal function after CI-AKI in rats with DM

Table 3 shows the renal function parameters evaluated. The mean serum creatinine level was higher in all three experimental groups than in the control group (*p* < 0.05). Our finding that inulin clearance was > 50% lower in the DMIC group than in the DM-only group is indicative of serious impairment of the glomerular filtration rate in the former. In addition, inulin clearance was higher in the DMICSH group than in the DMIC group (*p* < 0.05). Furthermore, urinary NGAL was lower in the DMICSH group than in the DMIC group (*p* < 0.05). Moreover, urinary albumin was than in the control group, as well as being higher in the DMIC group than in the DM-only group (DMIC: 10.80 ± 1.92; DM-only: 5.23 ± 2.80; and control: 1.06 ± 0.09, *p* < 0.05 for all). However, the mean TBARS level in the DMICSH group (0.78 ± 0.23) was lower than that observed for the DMIC and DM-only groups (*p* < 0.05). In addition, the mean thiol concentration (in nmol/mg total protein) was lower in the DM-only and DMIC groups than in the control group (DM-only: 14.97 ± 2.83; DMIC: 9.01 ± 2.90; and control: 26.48 ± 11.17, *p* < 0.05 for both), as depicted in Figure 2C. In the DMICSH group, the mean thiol concentration was 51.64 ± 18.06 nmol/mg, higher than the DMIC group value (*p* < 0.05).

**Table 3:** Renal function before and after CI-AKI, showing the effect of pretreatment with saline hydration.

Group	n	Serum creatinine(mg/dL) Mean ± SEM	Insulin clearance(mL/min) Mean ± SEM	Urinary NGAL(pg/mL) Mean ± SEM	Urinary albumin(ng/24 h) Mean ± SEM
ControI	7	0.31 ± 0.12	0.85 ± 0.13	42.2 ± 42.6	0.52 ± 0.27
DM-only	7	0.70 ± 0.07*	0.50 ± 0.12*	148.2 ± 37.7	2.89 ± 0.74*
DMIC	7	1.11 ± 0.18*, †	0.17 ± 0.02*,†	318.1 ± 52.6*,†	3.51 ± 0.91*
DMICSH	7	0.80 ± 0.03*,‡	0.32 ± 0.01V,*†‡	149.7 ± 42.2*‡	3.3 ± 0.35*

SEM, standard error of the mean.

* *p* < 0.005 vs. control.

† *p* < 0.005 vs. DM-only.

‡ *p* < 0.005 vs. DMIC.

## DISCUSSION

In this study, we have evaluated the renoprotective capacity of saline hydration against CI-AKI, considering DM as a risk factor. The pathophysiological mechanisms of CI-AKI include oxidative stress and vasoconstriction. Therefore, research on low-cost methods that promote renoprotection is still of great importance. We found that saline hydration protected renal function and hemodynamics, as well as maintaining kidney perfusion and mitigating oxidative stress damage, in animals with DM that received iodinated contrast.

Approximately 20% of individuals with DM develop diabetic nephropathy (i.e., have significant underlying kidney damage) 10–20 years after diagnosis. Among those receiving iodinated contrast, which is administered in various imaging tests employed in individuals with DM, the reported incidence of CI-AKI ranges from 5.7% to 24.9%^**(17–19)**^.

Although high-osmolar contrast media are no longer recommended in clinical practice, especially for high-risk patients such as those with DM, the present study aimed to elucidate the pathophysiological mechanisms of CI-AKI in this context. The objective was to demonstrate renal vulnerability to nephrotoxic insults using an experimental model that represents the gold standard for investigating this mechanism. Recent studies have shown features similar to those observed in the present study^**(4,9)**^.

In animals with DM, weight loss is one of the general indicators of metabolic regulation of the pathogenesis, because gluconeogenesis is stimulated to compensate for reduced glucose levels due to the unavailability of insulin, rendering the body unable to respond fully to insulin^**(20)**^. In addition, sustained hyperglycemia consists of a metabolic syndrome related to insulin resistance associated with hypertension, elevated LDL/triglycerides, and reduced HDL, with an increased risk of obesity and greater susceptibility to cardiovascular diseases^**(20,21)**^. In the present study, we observed polydipsia and polyphagia, which are related to cellular energy imbalance caused by sustained hyperglycemia, as well as polyuria resulting from hemodynamic changes driven by glomerular hyperfiltration. In this context, the thickening of the basement membrane promotes the accumulation of proteins in the extracellular matrix and the expansion of the mesangial matrix, thus promoting tubular growth and inducing disruption of the sodium/ glucose transporter in favor of the osmotic nature of water excretion^**(22)**^.

When exposed to high-osmolar contrast, tubular cells that are already deregulated by excess glucose suffer the insult of direct toxicity of iodinated contrast through the release of endothelin and vasoconstrictor prostaglandins that increase intracellular calcium levels and change cell polarity, thus inducing osmotic nephrosis^**(23)**^. In rats with DM followed for three months, urinary volume was found to increase, and that increase became more pronounced after a nephrotoxic drug was administered^**(9)**^. In the present study, we found that the metabolic changes induced by DM in combination with iodinated contrast administration were prevalent in the maintenance of kidney injury. However, saline hydration prevented the advancement of renal hypertrophy, as assessed by determining the kidney weight/body weight ratio. That phenomenon contributes to blocking protein accumulation in the extracellular matrix, as well as to mesangial matrix expansion and glomerular basement membrane thickening^**(20,22)**^.

Although numerous studies have investigated saline hydration and its adjuvants with the aim of preventing CIAKI in clinical practice^**(24,25)**^, few studies have assessed the hemodynamic mechanisms of the nephroprotective effect provided by various substances^**(4,11)**^. Our study demonstrated an increase in RVR and a decrease in RBF in rats with DM, both of which intensified in the rats with DM that received iodinated contrast, confirming the imbalance between vasoconstrictor and vasodilator substances caused by iodine toxicity. Therefore, the pathogenesis of CI-AKI associated with DM stands out due to constant hypoxia and the accumulation of hypoxia-inducible factor. These events can stimulate the renin-angiotensin system, enabling endothelin synthesis through prolonged hypoperfusion. This process results in the release of other vasomotor substances, such as adenosine, which further stimulates constriction of the renal arterioles and mesangial cells^**(6)**^. We found that saline hydration promoted renal perfusion, reducing RVR and increasing RBF in the animals that were induced to CI-AKI. Studies have demonstrated that oral or intravenous hydration before procedures involving the administration of nephrotoxic substances can be nephroprotective because of the action of vasodilators such as prostaglandin-1 and nitric oxide via nitrogen dioxide reduction, thus contributing to vasomotor balance^**(23,26)**^. Renal hypoperfusion is typically asymptomatic, rendering the diagnosis of CI-AKI particularly challenging—even among high-risk populations such as individuals with DM. Consequently, it is imperative that the multidisciplinary team remain vigilant in monitoring renal function, regulating the volume of contrast administered and implementing appropriate hydration protocols.

These preventive strategies aim to minimize the need for renal replacement therapy and to reduce the risk of progression to chronic kidney disease.

In the animals with DM in our study, serum creatinine and urinary NGAL were elevated, whereas inulin clearance (the gold standard for assessing the glomerular filtration rate) was reduced in those who received contrast. Overexpression of NGAL is related to intensive oxidative stress, and saline hydration was found to protect against such overexpression, as well as against the changes observed in other parameters. Elevated urinary NGAL has been used as an early biomarker of AKI because it has good specificity and sensitivity. It has been demonstrated that, in emergency departments, urinary NGAL is elevated within the first 3 h after kidney injury, thus preceding elevation of the creatinine concentration, which occurs 48–72 h after contrast infusion. Therefore, AKI care algorithms can be initiated earlier on the basis of urinary NGAL than on that of serum creatinine, and the former is considered an excellent predictor of clinical outcomes such as death and the need for dialysis after hospital admission^**(4,9)**^. Although urinary albumin was elevated in the animals with DM in our study, iodinated contrast administration had no effect on that variable, demonstrating that it is associated only with the pathogenesis of chronic DM^**(5)**^.

In the present study, oxidative stress was an important agent in the pathophysiology of CI-AKI. Among the animals with DM that received iodinated contrast, the data related to TBARS and urinary peroxides are indicative of intense oxidative injury. Depletion of adenosine triphosphate signals exacerbated oxygen consumption that results in medullary hypoxia, inducing lipid peroxidation, accumulation of cellular proteins via the hypoxia-inducible factor, and necrosis, as well as apoptosis of nuclear and mitochondrial DNA^**(20)**^. Therefore, reactive oxygen species are considered new biomarkers for oxidative injuries^**(27)**^. Studies have shown that increased hydrogen peroxide and superoxide are associated with worsening renal function^**(4,11,27)**^. However, an analysis of thiol levels by glutathione peroxidase inferred the consumption of the endogenous protective substrate of antioxidant enzymes. Numerous investigations have shown that redox imbalance plays a role in nephrotoxic kidney injury^**(4,9,11)**^. Our data show that saline hydration has an indirect antioxidant effect that prevents the generation of reactive oxygen species. The mechanism of that might involve increasing perfusion via greater RBF in the endothelium, which promotes the release of anti-inflammatory cytokines, thus minimizing oxidative damage^**(4,24)**^. In a study that assessed five hydration protocols for the prevention of cisplatin nephrotoxicity, saline hydration was found to be superior and to exhibit indirect antioxidant activity through a reduction in malondialdehyde levels^**(28)**^.

A comprehensive understanding of the molecular pathophysiology underlying CI-AKI supports evidencebased clinical decision-making for early diagnosis and facilitates the development of preventive strategies in highrisk patients, including the use of antioxidant approaches such as saline hydration, which can mitigate adverse renal outcomes. The clinical guidelines of the European Society of Urogenital Radiology recommend saline hydration as the primary preventive strategy for patients at increased risk of CI-AKI, such as those with DM. The standard protocol involves the administration of normal saline at 1 mL/ kg of body weight per hour, beginning at least 3–4 h before and continuing for 6 h after the procedure. In addition to hydration, other prophylactic measures are recommended, including minimizing the contrast dose, preferring low-osmolar contrast agents, and, when feasible, opting for diagnostic or therapeutic alternatives that do not require contrast media^**(29)**^. From a translational standpoint, our data underscore the importance of an individualized approach based on the identification of risk factors by a multidisciplinary team and contribute to the consolidation of saline hydration as an effective, highly reproducible measure for the prevention of CI-AKI.

Given the increase in the number of patients with comorbidities such as DM, which is associated with the need for interventional examinations involving contrast administration^**(3,6,30)**^, there is a need for data such as those from this study, which characterize saline hydration as a lowcost preventive measure with indirect antioxidant activity, as well as the capacity to reestablish renal hemodynamics. However, there is also a need for further studies to elucidate the molecular and cellular mechanisms of the saline hydration protocol.

## CONCLUSION

Numerous clinical studies have evaluated the nephroprotective effect of saline hydration in patients undergoing procedures involving the administration of iodinated contrast^**(8,25,31)**^. In this preclinical experimental study, we have elucidated the main mechanisms involved in saline hydration. We found that it preserved renal function, increasing inulin clearance by increasing RBF and reducing the redox imbalance.

## Data Availability

The data supporting the results of this study are published in the body of this article.
